# Sterol 14-alpha demethylase (CYP51) activity in *Leishmania donovani* is likely dependent upon cytochrome P450 reductase 1

**DOI:** 10.1371/journal.ppat.1012382

**Published:** 2024-07-11

**Authors:** Lindsay B. Tulloch, Michele Tinti, Richard J. Wall, Stefan K. Weidt, Victoriano Corpas- Lopez, Gourav Dey, Terry K. Smith, Alan H. Fairlamb, Michael P. Barrett, Susan Wyllie

**Affiliations:** 1 Wellcome Centre for Anti-Infectives Research, School of Life Sciences, University of Dundee, Dow Street, Dundee, United Kingdom; 2 Glasgow Polyomics, College of Medical, Veterinary and Life Sciences, University of Glasgow, Garscube Estate, Bearsden, Glasgow, United Kingdom; 3 Biomedical Sciences Research Complex, University of St Andrews, St Andrews, United Kingdom; 4 School of Infection & Immunity, College of Medical, Veterinary and Life Sciences, University of Glasgow, Glasgow, United Kingdom; University of Georgia, UNITED STATES OF AMERICA

## Abstract

Liposomal amphotericin B is an important frontline drug for the treatment of visceral leishmaniasis, a neglected disease of poverty. The mechanism of action of amphotericin B (AmB) is thought to involve interaction with ergosterol and other ergostane sterols, resulting in disruption of the integrity and key functions of the plasma membrane. Emergence of clinically refractory isolates of *Leishmania donovani* and *L*. *infantum* is an ongoing issue and knowledge of potential resistance mechanisms can help to alleviate this problem. Here we report the characterisation of four independently selected *L*. *donovani* clones that are resistant to AmB. Whole genome sequencing revealed that in three of the moderately resistant clones, resistance was due solely to the deletion of a gene encoding C24-sterol methyltransferase (*SMT1*). The fourth, hyper-resistant resistant clone (>60-fold) was found to have a 24 bp deletion in both alleles of a gene encoding a putative cytochrome P450 reductase (P450R1). Metabolic profiling indicated these parasites were virtually devoid of ergosterol (0.2% versus 18% of total sterols in wild-type) and had a marked accumulation of 14-methylfecosterol (75% versus 0.1% of total sterols in wild-type) and other 14-alpha methylcholestanes. These are substrates for sterol 14-alpha demethylase (*CYP51*) suggesting that this enzyme may be a *bona fide* P450R specifically involved in electron transfer from NADPH to CYP51 during catalysis. Deletion of *P450R1* in wild-type cells phenocopied the metabolic changes observed in our AmB hyper-resistant clone as well as in CYP51 nulls. Likewise, addition of a wild type *P450R1* gene restored sterol profiles to wild type. Our studies indicate that P450R1 is essential for *L*. *donovani* amastigote viability, thus loss of this gene is unlikely to be a driver of clinical resistance. Nevertheless, investigating the mechanisms underpinning AmB resistance in these cells provided insights that refine our understanding of the *L*. *donovani* sterol biosynthetic pathway.

## Author summary

The antifungal drug, amphotericin B, is also used in the treatment of visceral leishmaniasis, a potentially lethal parasitic disease infecting the specialised immune cells (macrophages) in the liver, spleen, and bone marrow. Treatment failures due to emerging drug resistance are a significant concern. Using a combination of genetic and biochemical approaches, we have confirmed the mechanisms by which these parasites become less sensitive to treatment with amphotericin B. In addition, we have identified a novel mechanism involving loss of a key enzyme (cytochrome P450 reductase 1) in the biosynthetic pathway to ergosterol, an important lipid component of the parasite’s plasma membrane. These studies increase our fundamental understanding of this important metabolic pathway and provide information that may be exploited to develop novel therapeutic strategies to combat this killer disease.

## Introduction

Leishmaniasis is a neglected tropical disease caused by infection with protozoan parasites of the *Leishmania* genus and transmitted through the bite of infected sandflies. There are an estimated 700,000–1,000,000 new cases annually, with the vast majority of infections occurring in the Americas, the Middle East, Central Asia, and East and West Africa [1]. Thus, leishmaniasis disproportionately affects some of the most impoverished parts of the world. Disease typically presents in three clinical forms depending upon the species of parasite responsible for the infection. The most common form is cutaneous leishmaniasis, which results in ulcers at the site of the sandfly bite that are self-healing but can leave life-long scars. Muco-cutaneous leishmaniasis, although not life-threatening, leads to partial or complete destruction of the mucous membranes of the nose, mouth, and throat. Visceral leishmaniasis (VL), resulting from systemic infection with *Leishmania donovani* or *L*. *infantum*, is the most severe form of the disease. It is characterised by bouts of fever, weight loss, anaemia, hepatosplenomegaly and, if left untreated, is usually fatal [1].

In the absence of a viable vaccine, treatment of the various forms of leishmaniasis is almost entirely reliant upon chemotherapy. At present, four drugs are in regular clinical use, namely pentavalent antimonials, miltefosine, paromomycin and amphotericin B (reviewed in detail in [2] and [3]). Treatment selection is based on a number of factors including parasite species, prevalence of resistance to specific drugs in the geographical area and available resources. Unfortunately, each one of these drugs suffers from issues that make them far from ideal including severe toxic side effects [4,5], acquired drug resistance [6] and prolonged treatment regimens [7]. Antimonials, such as sodium stibogluconate, have been used for the treatment of leishmaniasis since the 1940s but are associated with severe toxicity. While antimonials remain a front-line therapy for VL in East Africa, high levels of treatment failure associated with drug resistance now preclude the use of these drugs in India [8]. Miltefosine, the only orally bioavailable antileishmanial, is teratogenic and therefore cannot be prescribed to women of child-bearing age. Furthermore, the prolonged half-life of miltefosine (7 days) is considered to significantly increase the resistance potential of this alkylphosphocholine drug [2]. Indeed, 20% of patients treated with miltefosine during a cohort study of VL patients in Nepal relapsed [7], although a direct role for parasite resistance in these treatment failures was not established. Paromomycin was recently approved for the treatment of VL following successful phase II clinical trials in India [9,10], however, paromomycin performed less well in similar trials in Sudan [11,12].

Liposomal amphotericin B (AmB) is considered the standard of care for VL in many countries. Commonly, this polyene antibiotic is administered through multiple intravenous infusions (15 in total) over a 30-day period. The high cost of this treatment, coupled with associated toxicity, has driven the implementation of reduced treatment regimens [13]; however, there are concerns that these shortened regimes may be contributing to emerging amphotericin B resistance in India [14,15]. AmB belongs to a family of glycosylated macrolactone polyene antibiotics that demonstrate potent antifungal activity. The mechanism of action of these polyenes is thought to involve selective binding to ergostane-type rather than cholestane-type membrane sterols [16], leading to the formation of pores that disrupt cellular homeostasis and ultimately lead to cell death [17]. However, shorter polyene sterols thought to be incapable of pore-formation also display fungicidal activity leading to the suggestion that ergosterol sequestration rather than pore formation may be responsible for cell death [18,19]. One favoured model involves AmB forming extra-membranous sponge-like aggregates that extract ergosterol from lipid bilayers [20,21]. The selective toxicity of AmB for fungi and *Leishmania* may depend on the different extraction rates for ergosterol and related sterols in the membranes of these pathogens, in contrast to mammalian cells where cholesterol is the major sterol [22].

**Fig 1:**
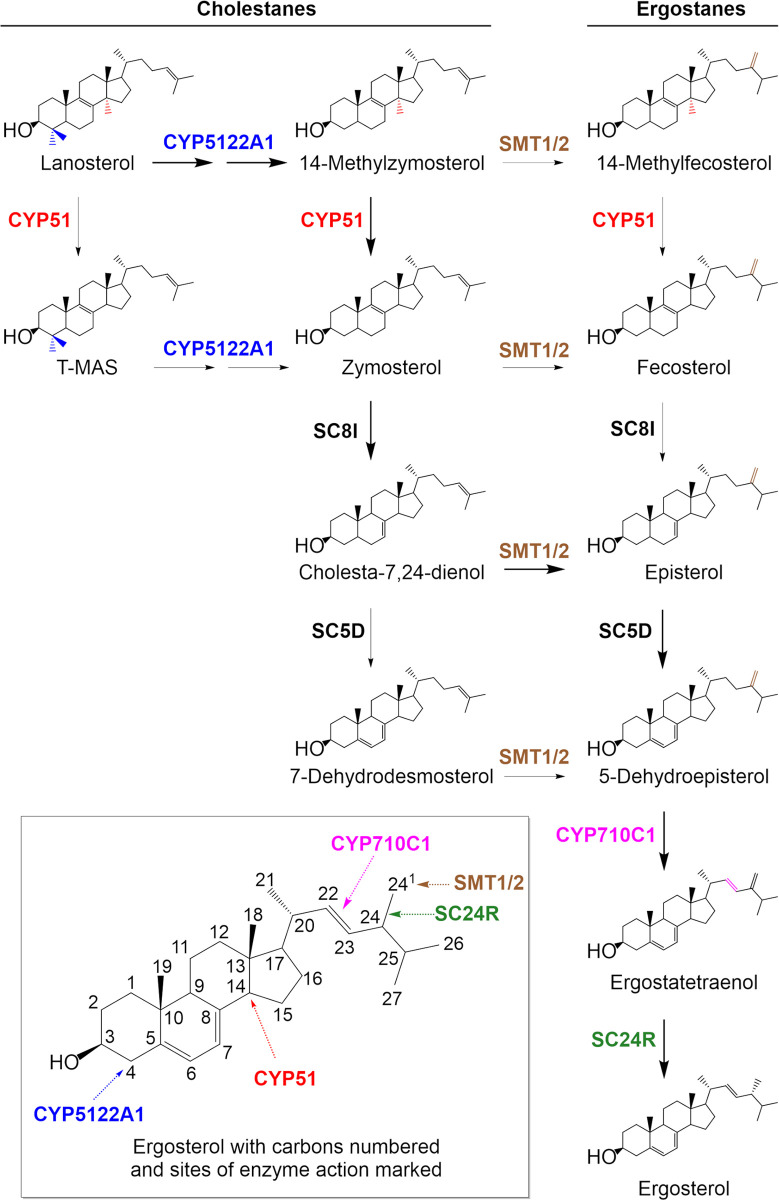
*Leishmania* sterol biosynthetic pathway.

Multiple studies in both fungi and *Leishmania* have linked a plethora of mutations in the enzymes of the ergosterol biosynthetic pathway to AmB resistance (**Fig 1**) [23–26]. Many of the mutations are thought to reduce the levels of ergosterol in the membranes of these organisms. Altered antioxidant defences have also been associated with modest resistance [26–28] suggesting that oxidative stress may also play a role in the cytocidal activity of this polyene macrolide. Indeed, previous studies have demonstrated that AmB has the ability to auto-oxidise [29]. Collectively, these studies indicate that the mechanisms of action and mechanisms underpinning AmB resistance may be more complex than generally accepted. For drugs used in a clinical setting, it is vital that there is a comprehensive understanding of the full range of such mechanisms. This knowledge can be used not only to inform the selection of the best possible partner drugs for future combination therapies but also to prioritise the development of drugs capable of overcoming existing clinical resistance. Here we report the characterisation of AmB-resistant *L*. *donovani* clones generated through *in vitro* selection. Whole genome sequencing revealed that in three moderately resistant clones (5-8-fold compared to wild-type), resistance was due solely to the deletion of the gene encoding C24-sterol methyltransferase (*SMT1*). A fourth clone demonstrating the most significant levels of AmB resistance (>60-fold compared to wild-type) was found to be functionally null for a putative P450 reductase. Sterol profiling indicated that *P450R1* null parasites were deficient in sterol 14-alpha demethylase (CYP51) activity leading us to hypothesise that this putative reductase may be responsible for regeneration/reduction of CYP51. To our knowledge this represents the first time this putative P450R, which we now call P450 reductase 1 (P450R1), has been functionally characterised and implicated in AmB resistance. These studies expand our current understanding of the sterol biosynthetic pathway of *L*. *donovani*.

## Results and discussion

### Resistance generation followed by whole genome sequencing

**Fig 2:**
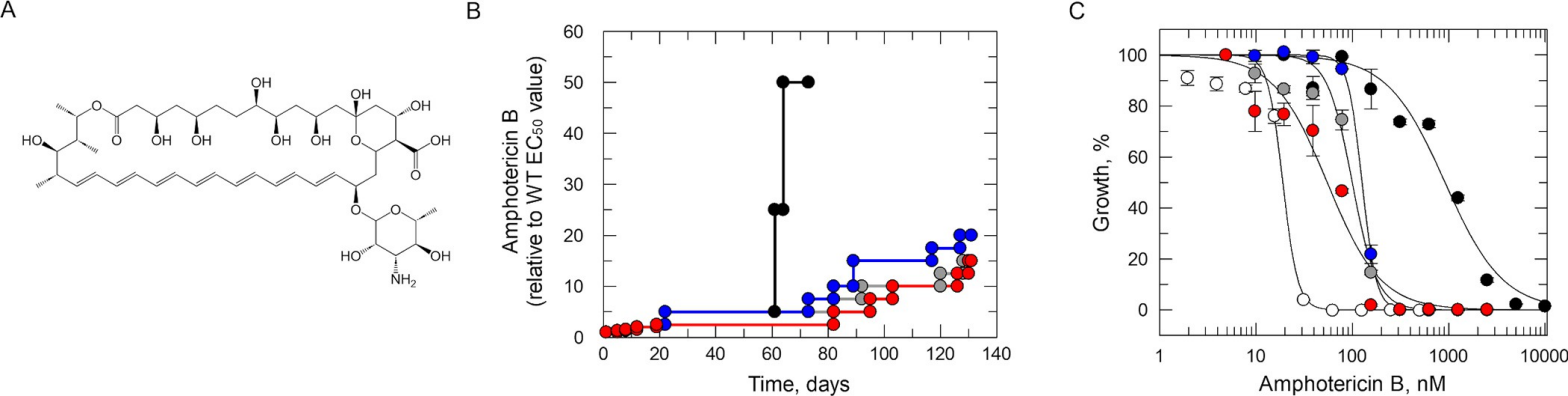
*In vitro* evolution of AmB resistance in *L*. *donovani*. (A) Chemical structure of amphotericin B. (B) Schematic representation of the generation of AmB-resistant cell lines in *L*. *donovani*. Each passage of cells in culture (circles, lines 1–4) is indicated with cell lines 1–4 indicated in black, grey, blue, and red, respectively. (C) Dose-response EC_50_ values for AmB were determined for WT (white) and cloned resistant cell lines 1–4 (black, grey, blue, and red, respectively). These representative curves are the nonlinear fits of data using a two-parameter EC_50_ equation provided by GraFit. An EC_50_ value of 19 ± 2 nM was determined for AmB against WT promastigotes. EC_50_ values for resistant clones AmB R1–4 were 915 ± 118, 100 ± 8, 126 ± 0.8 and 55 ± 8 nM, respectively. These EC_50_ values represent one biological replicate, composed of two technical replicates. Collated datasets reporting the weighted mean ± SD of multiple biological replicates are summarised in **Table 1**.

*L*. *donovani* promastigotes resistant to AmB were selected through *in vitro* evolution (**Fig 2**). Starting at 20 nM (1× EC_50_), four drug-sensitive, clonal cell lines were exposed to stepwise increasing concentrations of AmB until they could grow at concentrations equivalent to >20× the established EC_50_ value (**Fig 2B**). The four independently generated resistant cell lines were cloned by limiting dilution, and the susceptibility of the resulting clones to AmB was assessed. Three of the clones (AmB R2, 3 and 4) were between 5- and 8-fold less sensitive to AmB than the wild-type (WT) parental clone (**Fig 2C** and **Table 1**). However, AmB R1 demonstrated considerably higher levels of resistance at >60-fold less susceptible to the drug. In each case, resistance demonstrated by each clone was found to be relatively stable over at least 30 passages in culture in the absence of compound (**Table 1**).

**Table 1:** Collated AmB EC_50_ values for WT, resistant and transgenic promastigote cell lines.

Cell line	EC_50_ value, nM	Fold shift (relative to WT)	Biological replicates
WT	19 ± 0.1	-	8
AmB R1	1200 ± 91	63	8
AmB R2	148 ± 5	8	3
AmB R3	152 ± 1	8	8
AmB R4	88 ± 5	5	3
AmB R1 (p30)	816 ± 38	43	2
AmB R2 (p30)	107 ± 9	6	2
AmB R3 (p30)	181 ± 4	10	2
AmB R4 (p30)	88 ± 3	5	2
AmB R3 + SMT1^WT^	28 ± 1	1	4
AmB R3 + SMT2^WT^	33 ± 2	2	4
SMT1 SKO	36 ± 1	2	5
SMT1 DKO	143 ± 7	8	5
SMT2 DKO	31 ± 1	2	4
SMT1/2 DKO	187 ± 4	10	8
SMT1/2 DKO + SMT1^WT^	16 ± 0.01	1	3
SMT1/2 DKO + SMT2^WT^	23 ± 1	1	3
AmB R1 + P450R1^WT^	42 ± 3	2	4
AmB R1 + P450R1^MUT^	1880 ± 150	99	3
P450R^Δ605–612^	1000 ± 65	53	6
P450R DKO	1211 ± 55	64	8
P450R DKO + P450R1^WT^	26 ± 1	1	3
P450R DKO + P450R1^MUT^	2758 ± 196	145	3
CYP51 DKO	3520 ± 240	185	8

*EC_50_ values represent the weighted mean ± standard deviation of the indicated number of biological replicates with each biological replicate comprised of at least two technical replicates.

**Fig 3:**
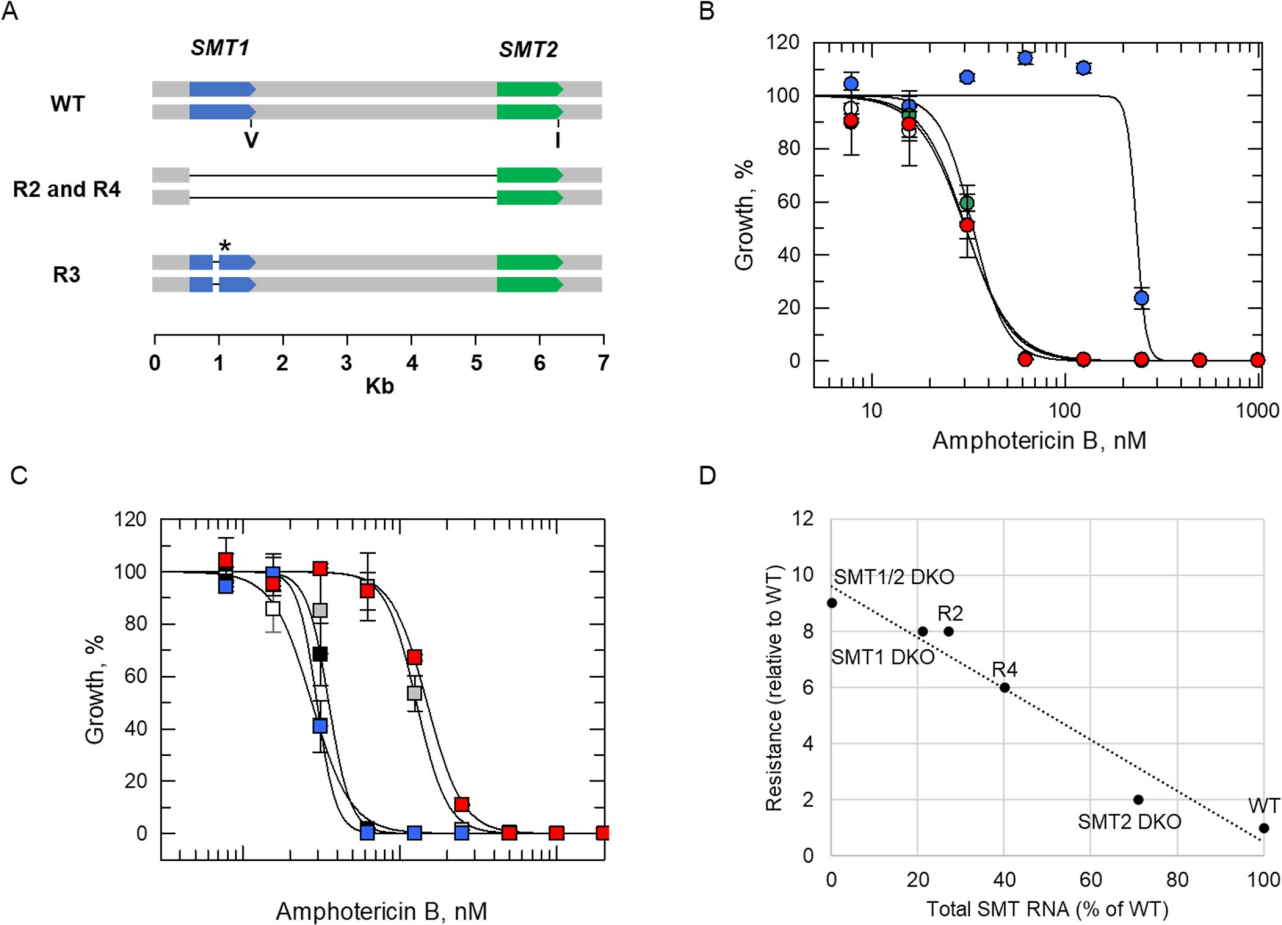
Investigating the impact of *SMT1* and *SMT2* deletions on AmB susceptibility. (A) Schematic representation of *SMT1*-related deletions identified in AmB R2–4 cell lines. The site of the single amino acid change between SMT1 (V indicating valine) and SMT2 (I indicating isoleucine) are shown. The site of the new stop codon in AmB R3 is denoted by an asterisk. (B) Dose-response curves for WT (white), AmB R3 (blue), AmB R3 plus *SMT1*^*WT*^ add-back (green) and AmB R3 plus *SMT2*^WT^ add-back (red) clones treated with AmB. EC_50_ values of 30 ± 1, 234 ± 36, 33 ± 2 and 31 ± 1 nM were determined for WT, AmB R3, AmB R3 plus *SMT1*^WT^ add-back and AmB R3 plus *SMT2*^WT^ add-back promastigotes, respectively. (C) Dose-response curves for WT (white), *SMT1* SKO (black), *SMT1* DKO (grey), *SMT2* DKO (blue), and *SMT1/2* DKO (red). EC_50_ values of 28 ± 1, 35 ± 1, 128 ± 6, 30 ± 0.6 and 149 ± 4 nM were determined for WT, *SMT1* SKO, *SMT1* DKO, *SMT2* DKO, and *SMT1/2* DKO promastigotes, respectively. These EC_50_ curves and values represent one biological replicate, composed of two technical replicates. Collated datasets reporting the weighted mean ± SD of multiple biological replicates are summarised in **Table 1**. (D) Plot of total *SMT* RNA versus level of AmB resistance, relative to WT.

Genomic DNA recovered from the four resistant clones was analysed by whole genome sequencing (WGS) (**S1 Table**). The three cell lines demonstrating comparatively modest levels of resistance to AmB (AmB R2–4) were all found to maintain an additional copy of chromosome 26, compared to the parental WT clone (**S2 Table**). Perhaps most notably, all three clones possessed deletions within the same sterol C24-methyl transferase (SMT) locus previously associated with AmB resistance [25, 26]. This locus is comprised of a tandem array of two near-identical transferase genes, *SMT1* (LdLV9.36.2.209980) and *SMT2* (LdLV9.36.2.209990) that differ by a single amino acid at position 321 (valine in SMT1 and isoleucine in SMT2). Sequence analysis enabled us to confirm that both copies of *SMT1*, as well as the intergenic region between *SMT1* and *2* genes, were deleted from cell lines AmB R2 and R4 (**Fig 3A, S3 Table**). In AmB R3, a homozygous 17-bp deletion was identified that introduced a premature stop codon into *SMT1*. As a representative of our *SMT1* deletion mutants, AmB R3 promastigotes were differentiated into axenic amastigotes and found to retain their AmB-resistant phenotype in this more medically relevant, mammalian stage of the parasite (**S4 Table**).

**Fig 4:**
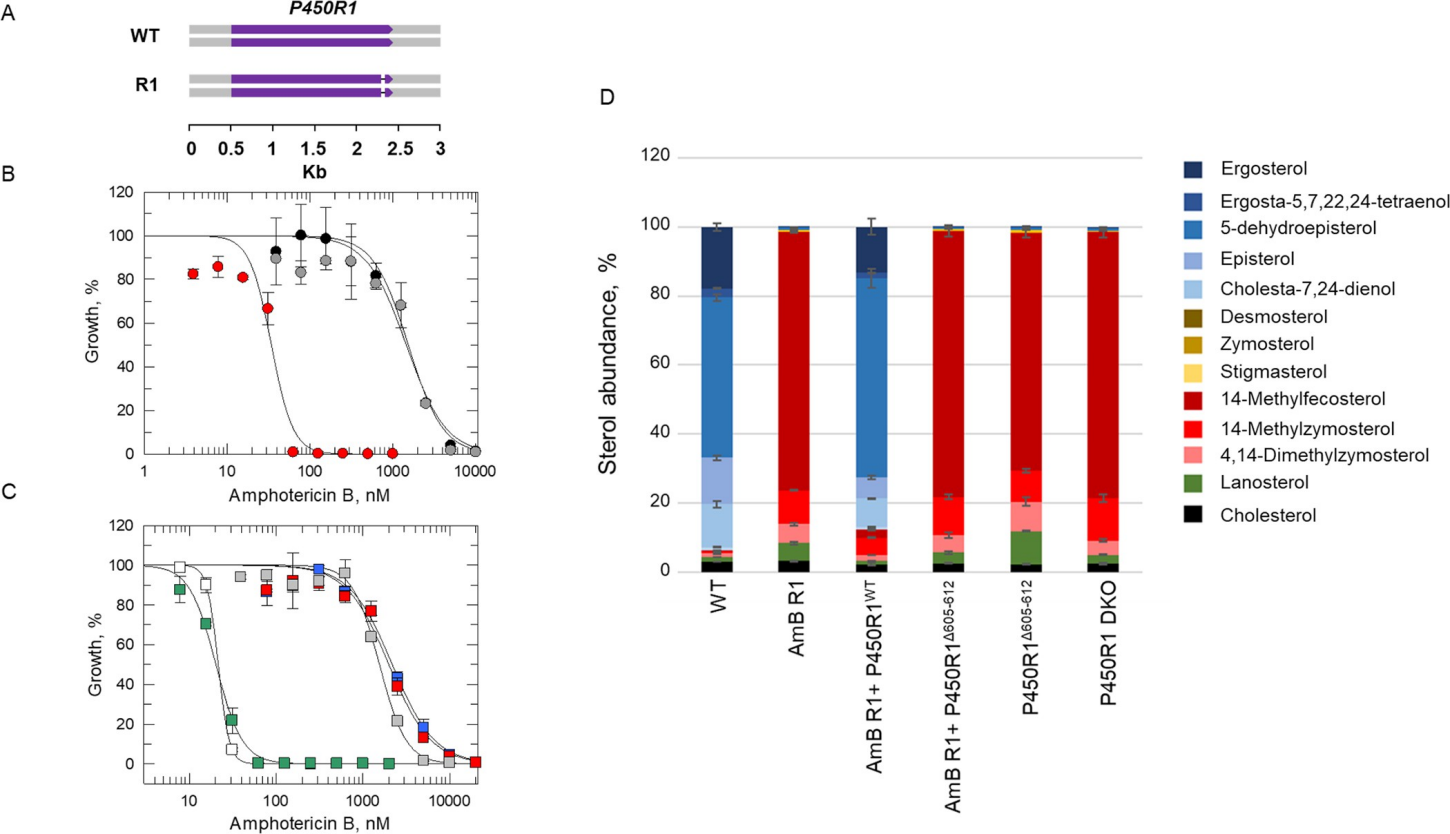
Investigating the impact of P450R1 functional loss on AmB susceptibility and sterol composition. (A) Schematic representation of the homozygous 24-bp deletion within *P450R1* in the AmB R1 cell line. (B) Dose–response curves for AmB R1 (grey), AmB R1 plus *P450R1*^WT^ add-back (red) and AmB R1 plus *P450R1*^Δ605–612^ add-back (black) promastigote clones treated with AmB. EC_50_ values of 1530 ± 116, 34 ± 3.9, and 1450 ± 211 nM were determined for AmB R1, AmB R1 plus *P450R1*^WT^ add-back, and AmB-R1 plus *P450R1*^Δ605–612^ addback, respectively. (C) Dose-response curves for WT (white), P450R1 DKO (blue), *P450R1* DKO plus *P450R1*^*WT*^ add-back (green), *P450R1* DKO plus *P450R1* ^Δ605–612^ add-back (red) and *P450R1*^*Δ605–612*^ (grey) promastigotes treated with AmB. EC_50_ values of 22 ± 0.1, 2170 ± 204, 20 ± 0.7, 1990 ± 193 and 1550 ± 99 nM were determined for WT, *P450R1* DKO, *P450R1* DKO plus *P450R1*^*WT*^ add-back, *P450R1* DKO plus *P450R1*^Δ605–612^ add-back and *P450R1*^Δ605–612^ promastigotes, respectively. These EC_50_ curves and values represent one biological replicate, composed of two technical replicates. Collated datasets reporting the weighted mean ± SD of multiple biological replicates are summarised in **Table 1**. (D) Sterol profiling of WT and *P450R1* mutant promastigotes. Values are the mean ± SD from biological replicates.

In contrast, the hyper-resistant clone AmB R1 maintained a full complement of *SMT1* and *2* genes (**S3 Table**). CNV analysis confirmed that this clone lost a copy of chromosome 22, reducing the chromosome level from tetraploid to triploid (**S2 Table**). We reasoned that reducing the dosage of genes on chromosome 22 by 25% would be unlikely to drive a >50-fold shift in drug susceptibility. AmB R1 maintained a total of eight mutations (**S1 Table**). Five of these mutations were shared with one or more of the cell lines demonstrating modest AmB-resistance and therefore deemed unlikely to be responsible for hyper-resistance in AmB R1. Three mutations unique to AmB R1 were identified including a heterozygous 10-bp deletion in a gene (LdBPK_312290.1) encoding a hypothetical protein unique to *Leishmania spp*, a 69-bp heterozygous insertion within the gene (LdBPK_360990.1) encoding the 40S ribosomal protein S18 and a homozygous 24-bp deletion within a gene (LdBPK_281350.1) encoding a putative cytochrome P450 reductase (also known as a haemoprotein reductase, P450R1) (**Fig 4A**). Since P450Rs, are known to play a key role in sterol biosynthesis and drug metabolism [30], we sought to further investigate the role of this INDEL in AmB hyper-resistance alongside the role of *SMT1* deletion in moderate resistance.

### Investigating the role(s) of SMT1 and SMT2 in AmB resistance

SMTs catalyse the methylation of the C24 side chain of cholestanes to form the cognate ergostane (**Fig 1**). Broadly speaking, organisms that maintain both SMT1 and SMT2 enzymes produce both C24-methylated and -ethylated sterols while those possessing only SMT1 produce C24-methylated sterols. Expression of *Nicotiana tabacum* SMT1 in SMT1-deficient *Saccharomyces cerevisiae* resulted in the production of C24-methylated sterols, while expression of SMT2 in these SMT1-deficient yeast resulted in the production of C24-ethylated sterols [31]. Thus, SMT1 is capable of catalysing the addition of a single methyl group while SMT2 can catalyse sequential methylation of sterols [32]. Dissection of the individual roles of *SMT1* and *SMT2* gene products in *Leishmania* has not yet been ascertained, but the conservation of the two genes across *Leishmania species* indicates they have distinct functions.

To interrogate the role of SMTs in AmB resistance, multiple transgenic cell lines were generated. In the first instance, we focused on the role of *SMT1* deletion in the modest resistance demonstrated by AmB R2–4. An ectopic copy of *SMT1* was re-introduced into AmB R3 via the *Leishmania*-specific expression vector pIR1 [33]. Successful re-introduction of SMT1 into AmB R3 was confirmed by quantitative proteomics (**S3A Fig**) and found to restore AmB-sensitivity to these formerly resistant promastigotes (**Fig 3B, Table 1**). In addition, sequential knock-out of *SMT1* from WT cells by CRISPR-Cas9 gene editing, confirmed by WGS (**S3 Table**), resulted in promastigotes (*SMT1* DKO) that were 8-fold less susceptible to AmB, a similar level of resistance demonstrated by AmB R2–4. *SMT1* DKO promastigotes readily differentiated into axenic amastigotes and remained resistant to AmB in this developmental form (**S4 Table**). Collectively, these data confirm the causal link between SMT1 functional loss and AmB resistance in our moderately resistant *L*. *donovani* promastigote cell lines and are entirely consistent with previous observations in *L*. *mexicana* [25,26], and some earlier reports in *L*. *donovani* [14,34–36].

Since SMT1 and 2 are virtually identical, except for of a single amino acid substitution, we investigated the potential role of SMT2 in AmB susceptibility and/or resistance. In the first instance, we overexpressed SMT2 in AmB R3 (**S3 Fig**). Overexpression of this putative methyltransferase reverted AmB resistance in R3 promastigotes (**Table 1**) and axenic amastigotes (**S4 Table**) indicating that SMT2 can functionally complement SMT1. Next, both gene copies of *SMT2* were deleted from WT parasites via CRISPR-Cas9 gene editing, with deletion confirmed by WGS (**S3 Table**). However, removal of both *SMT2* gene copies from WT had little or no impact on levels of AmB resistance (**Tables 1, S4 and Fig 3C**). A ransgenic cell line was then generated where both *SMT1* and *2* genes were simultaneously deleted (*SMT1/2* DKO). Deletion of both *SMT1* and *2* did not markedly affect the growth rate of the resulting transgenic cell line confirming that both transferases are not required for either *L*. *donovani* promastigote or axenic amastigote viability. The resulting *SMT* null cell line demonstrated only marginally enhanced AmB resistance compared to our *SMT1* double knock-out (*SMT1* DKO) parasites (AmB susceptibility reduced by 10-fold versus 8-fold relative to WT).

Previous studies have reported that *SMT1* RNA levels are substantially higher than those of SMT2 in *L*. *donovani* [34] and *L*. *mexicana* [25]. Using quantitative RT-PCR, total *SMT* transcript levels in WT, *SMT1* and *SMT2* DKO cell lines were determined and compared (**S1 Fig**). In SMT1 null promastigotes, total *SMT* transcript levels were ~70% lower than measured in WT cells while transcript levels in SMT2 null parasites were ~30% lower. Prompted by these observations, we then measured *SMT* transcript levels in resistant cell lines (AmB R2–4) and plotted against AmB resistance (relative to WT). Levels of AmB resistance were found to inversely correlate to *SMT* transcript levels in these clones with an R^2^ value of 0.95 (**Fig 3D**). Thus, modulating overall SMT enzyme activity can directly impact AmB susceptibility in *L*. *donovani*. Our data also indicates that SMT1 is more highly expressed and is likely the dominant SMT, thus explaining the profound impact of *SMT1* deletion on AmB resistance compared to *SMT2* deletion. Subsequent quantitative proteomics analysis of SMT levels in *SMT1* and *SMT2* DKO promastigotes confirm this observation with SMT1 expression levels again higher than SMT2 (**S3A Fig**).

Previous studies in *L*. *donovani* have demonstrated that while promastigotes synthesise only ergosterol (C24-methylated), axenic amastigotes synthesise both ergosterol and stigmasterol (C24-ethylated) [37]. This led us to hypothesise that, as is the case in other organisms, SMT2 may be responsible for synthesis of C24-ethylated sterols and that SMT2 may be predominantly expressed in the more medically relevant amastigote stage of the parasite. However, quantitative RT-PCR analysis of total *SMT* transcript levels in *SMT1* and *2* null axenic amastigotes revealed that SMT1 accounts for >60% of the total *SMT* transcripts in these parasites (**S2 Fig**), essentially replicating our promastigote data, with quantitative proteomics analysis confirming this observation at the protein level (**S3A Fig**). In addition, *SMT2* null axenic amastigotes remain susceptible to AmB (**S4 Table**). It is possible that the observed low levels of SMT2 expression are sufficient to account for the C24-ethylated stigmasterol previously detected in amastigotes [37] but under these circumstances we would also expect to detect stigmasterol in promastigotes. While SMT2 expression levels are lower than SMT1, particularly in axenic amastigotes (**S3B Fig**), the fact that we can detect SMT2 expression and that overexpression can complement for SMT1 loss at both stages of the parasite, confirms that *SMT2* encodes a functional methyltransferase. We acknowledge that axenic amastigotes are not a perfect model for this critical stage of the parasite lifecycle and future studies will focus on measuring SMT expression levels in more physiologically relevant amastigotes recovered from infected macrophages.

### Investigating the association between P450R1 and AmB hyper-resistance

To determine if loss of P450R1 full length expression plays a direct and/or significant role in the hyper-resistant phenotype of AmB R1, an ectopic copy of this reductase was reintroduced into the cell line. Successful expression of P450R1 in these resistant parasites was confirmed by quantitative proteomics (**S3C Fig**). Adding back this functional copy of *P450R1* restored AmB susceptibility to almost WT levels. However, introducing an ectopic copy of *P450R1* bearing the 24-bp INDEL (Δ605–612) identified in AmB1 parasites failed to rescue drug susceptibility (**Table 1, Fig 4B**). Next, we utilised CRISPR-Cas9 gene editing to replicate the *P450R1* homozygous INDEL in WT promastigotes. Successful deletion of the 24-bp from both copies of *P450R1* was confirmed through Sanger sequencing, and the resulting clones were assessed to establish their susceptibility to AmB. Introduction of this INDEL into WT parasites induced hyper-resistance to AmB at a similar level to that demonstrated in our AmB R1 cell line (53-fold versus 63-fold shift, respectively, **Table 1, Fig 4C**). These data suggest that mutation of P450R1 is likely the primary driver for AmB hyper-resistance in AmB R1. To our knowledge, this represents the first time that a P450R has been implicated in resistance mechanisms to this clinical anti-microbial agent in either *Leishmania* or fungi.

We hypothesised that the 24-bp INDEL may have ablated P450R1 activity in AmB R1. To test this, we generated a *P450R1* null cell line using CRISPR-Cas9 gene editing. The resulting transgenic parasites were hyper-resistant to AmB and at a similar magnitude to our AmB R1 cell line, with EC_50_ values shifting 64-fold relative to WT (**Table 1, Fig 4C**). These data confirm that P450R1 is not essential in *L*. *donovani* promastigotes and indicate that AmB R1 is almost certainly a functional null for P450R1. Notably, AmB R1 and P450R1 null cell lines could not be differentiated into axenic amastigotes, while both lines bearing a P450R1^WT^ add-back could. In contrast, cell lines complemented with P450R1^Δ605–612^ were unable to differentiate. We also assessed the ability of these cell lines to sustain an infection within primary macrophages. AmB R1, *P450R1* DKO and add-back cell lines were grown in culture until they reached metacyclic promastigote stage. The resulting parasites were incubated with starch-elicited mouse peritoneal macrophages for 12 h. Non-phagocytosed promastigotes were removed and infected macrophages incubated for 72 h. The ability of AmB R1 and *P450R1* DKO cell lines to establish and sustain an infection in primary macrophages was severely compromised, as determined by comparing the mean numbers of amastigotes per infected macrophage compared to WT (**S5 Fig**). Adding back a functional copy of *P450R1* to DKO parasites restored infectivity to wild-type levels. However, addback of this gene did not restore infectivity to AmB R1. We hypothesise that this discrepancy may be due to P450R1-independent changes that may have occurred within these parasites during prolonged resistance selection *in vitro*. Nevertheless, these data suggest that P450R1 is essential for both *L*. *donovani* amastigote viability and infectivity. Reassuringly, they also indicate that the hyper-resistant phenotype associated with AmB R1 could not be replicated in the clinic.

### Analysis of sterol composition in AmB-resistant and transgenic promastigotes

It is widely accepted that the mechanism of action of AmB is principally through direct binding to, and sequestration of, ergosterol or related ergostane-type sterols [16,21,38]. Moreover, a multitude of studies have associated changes in sterol composition to AmB resistance in several organisms [25,26,34,39]. Since the impact of SMT1 deletion on sterol composition has already been thoroughly investigated in *L*. *mexicana* [26], here we focused on profiling sterol changes induced by deletion of P450R1. Sterols within WT, AmB R1 and transgenic cell lines were profiled using gas chromatography-mass spectrometry (GS-MS) and identified based on retention time and spectral matches to established standards alongside comparison to the literature values (**S6 Table**). The predominant sterols detected in our *L*. *donovani* WT promastigotes were 5-dehydroepisterol (46.5 ± 0.9%), ergosterol (17.8 ± 1.0%), episterol (13.5 ± 0.7%) and cholesta-7,24-dienol (12.4 ± 0.8%), all ergostane-type sterols produced at the end of the *Leishmania* sterol biosynthetic pathway (**Figs 1**, **4D, and S7 Table)**. In contrast, vanishingly small quantities of these ergostane-type sterols were detected in AmB R1. Instead, our hyper-resistant parasites were enriched in 14-methylated sterols produced earlier in the biosynthetic pathway, namely C14-methylfecosterol (75.1 ± 0.4%), C14-methylzymosterol (9.9 ± 0.1%) and 4,14-dimethylzymosterol (5.5 ± 0.4%). As expected, introducing a functional copy of *P450R1* back into AmB R1 promastigotes was sufficient to restore WT sterol composition, while add back of *P450R1* bearing the previously described INDEL could not. The sterol profiles of the *P450R1* DKO cell line, as well as the CRISPR-edited P450R1^Δ605–612^ cell line, closely matched the profile of AmB R1 providing further evidence that deletion of amino acids 605–612 results in loss of P450R1 function.

### Assessing the functional role of P450R1 in sterol biosynthesis

The loss of ergostane-type and accumulation of 14-methylated sterols in our various *P450R1* null cell lines matches sterol profiles previously reported for *CYP51* null *L*. *major* promastigotes [40], as well as promastigotes treated with azoles known to specifically inhibit CYP51 [41]. These observations led us to hypothesise that P450R1 may be responsible for regenerating CYP51 catalytic capacity in *L*. *donovani*. In contrast to human cells, which maintain a single NADPH-dependent cytochrome P450 reductase, three putative cytochrome P450 reductases have been identified in *L*. *donovani*. The focus of our current study, P450R1 and P450R2 (LdBPK_352600.1) share 35% sequence identity and 35% and 24% with human P450R, respectively. The remaining putative leishmanial P450R (P450R3, LdBPK_342500.1) more closely resembles the human NADPH-dependent diflavin oxidoreductase 1 (NDOR1), a central component of the cytosolic iron-sulphur (Fe-S) protein assembly machinery [42]. To our knowledge, specific functions have yet to be assigned to the three putative cytochrome P450 reductases in *Leishmania*. Alongside these reductases, *Leishmania* spp. maintain three cytochrome P450s (CYP51, CYP5122A1 and CYP710C1) that act at different points in the sterol biosynthetic pathway (**Fig 1**). It is tempting to suggest that the three P450Rs have specific roles, regenerating specific CYPs.

**Fig 5:**
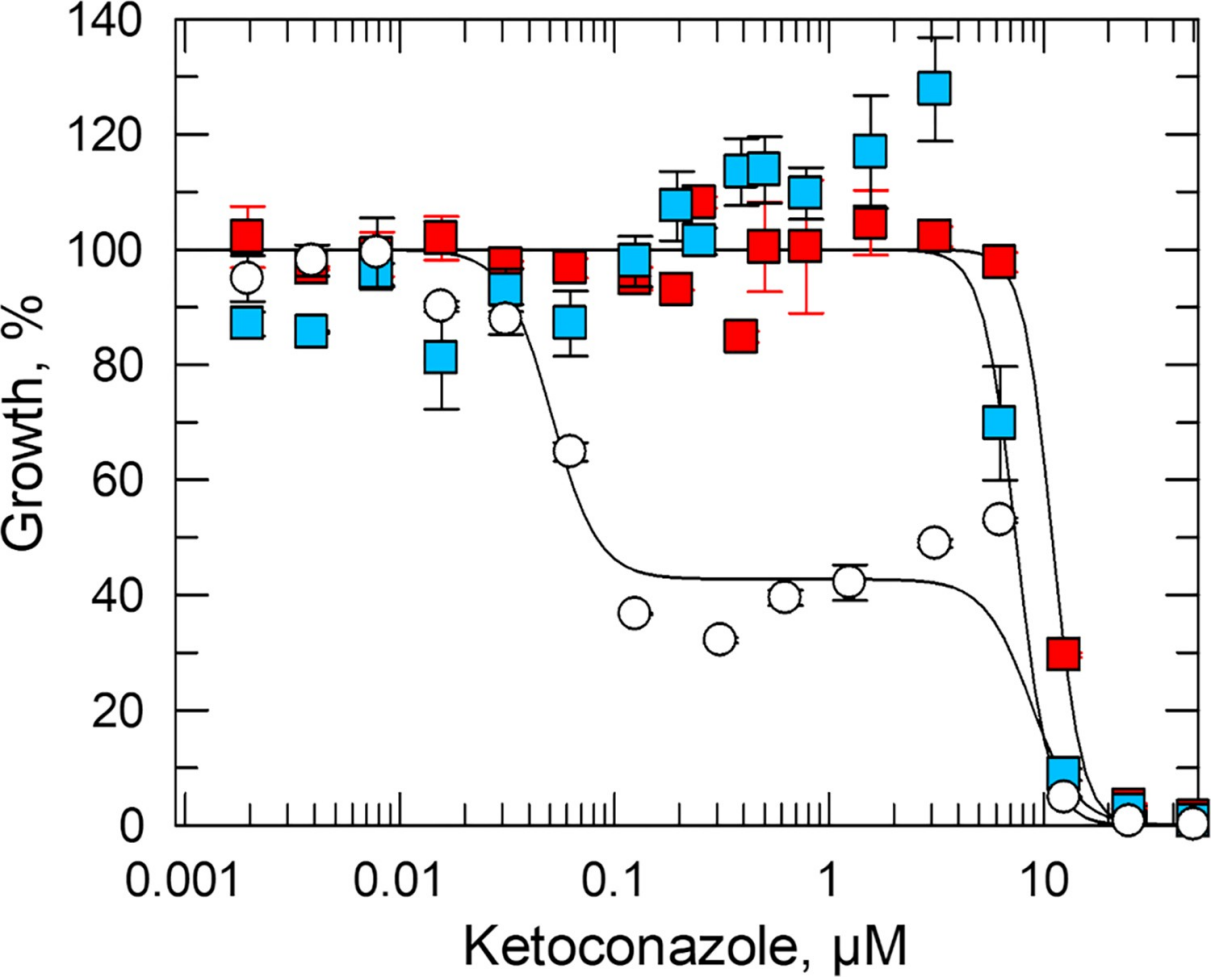
Investigating the impact of P450R1 and CYP51 functional loss on ketoconazole susceptibility. Dose–response curves for WT (white), CYP51 DKO (blue) and P450R1 DKO (red) promastigote clones treated with ketoconazole. EC_50_ values of 0.03 ± 0.01 (lower) and 10 ± 8 μM (upper) were determined for WT promastigotes while values of 11 ± 3 and 7 ± 1 μM were determined for P450R1 DKO and CYP51 DKO parasites, respectively. These EC_50_ curves and values represent one biological replicate, composed of two technical replicates. Collated datasets reporting the weighted mean ± SD of multiple biological replicates are summarised in **S8 Table**.

To explore the apparent association between P450R1 and CYP51, a *L*. *donovani CYP51* DKO cell line was engineered. While knock-out of *CYP51* has been achieved in *L*. *major* [40] with the resulting parasites capable of infecting mice, previous attempts to generate CYP51-null *L*. *donovani* were unsuccessful [43]. This led to speculation that CYP51 is indispensable in *L*. *donovani* promastigotes and that CYP51-directed therapies should be considered for visceral leishmaniasis [43]. Here, we were able to delete both copies of the CYP51 encoding gene in a single round of CRISPR-Cas9 gene editing. Successful removal of both gene copies was confirmed by WGS. The resulting *L*. *donovani* promastigotes did grow slower than WT but could differentiate into axenic amastigotes and were able to infect mouse peritoneal macrophages, albeit at a lower level than WT (**S5 Fig**). These findings confirm that CYP51 is not essential for survival of *L*. *donovani*, at least *in vitro*, and that azoles known to target this enzyme should not be considered for the treatment of visceral leishmaniasis. Consistent with previous reports in *L*. *major* [40], CYP51 null *L*. *donovani* promastigotes were hyper-resistant to AmB (**Table 1**). Next, we determined the sensitivity of our WT and (*P450R1* and *CYP51*) DKO cell lines to the established CYP51 inhibitor ketoconazole [44]. Drug susceptibility assays with WT promastigotes exposed to ketoconazole resulted in a pronounced biphasic EC_50_ curve with a lower EC_50_ value of 29 ± 5 nM and upper curve value of 5 ± 0.5 μM (**Fig 5, S8 Table**). Previous studies by Xu and colleagues proposed that lower concentrations of ketoconazole (nM) are cytostatic for *L*. *major* promastigotes due to inhibition of non-essential CYP51, but higher concentrations (>2 μM) are cytocidal through inhibition of an as yet unidentified secondary target [40]. In support of this hypothesis, ketoconazole treatment of our *CYP51* nulls resulted in a canonical sigmoidal dose response curve (EC_50_ value– 5 ± 0.6 μM) rather than a biphasic response. Notably, the response of P450R1-deficient promastigotes (**Fig 5** and **S8 Table**) to ketoconazole exposure closely mimicked that of the *CYP51* nulls. Collectively, these data demonstrate that CYP51 and *P450R1* DKO cell lines phenocopy in their responses to AmB and ketoconazole and are consistent with our hypothesis that P450R1 may be required for CYP51 activity.

The discrepancy in the essentiality between CYP51 and P450R1 indicates that P450R1 has functions in addition to its role in regeneration of CYP51 that are essential for amastigote viability. Indeed, P450R, also known as haemoprotein reductases, are likely to be involved in other redox functions. Future studies will aim to comprehensively characterise all functions of P450R1 as well as the other *Leishmania* P450 reductases.

### Structural implications of the P450R1 INDEL

**Fig 6:**
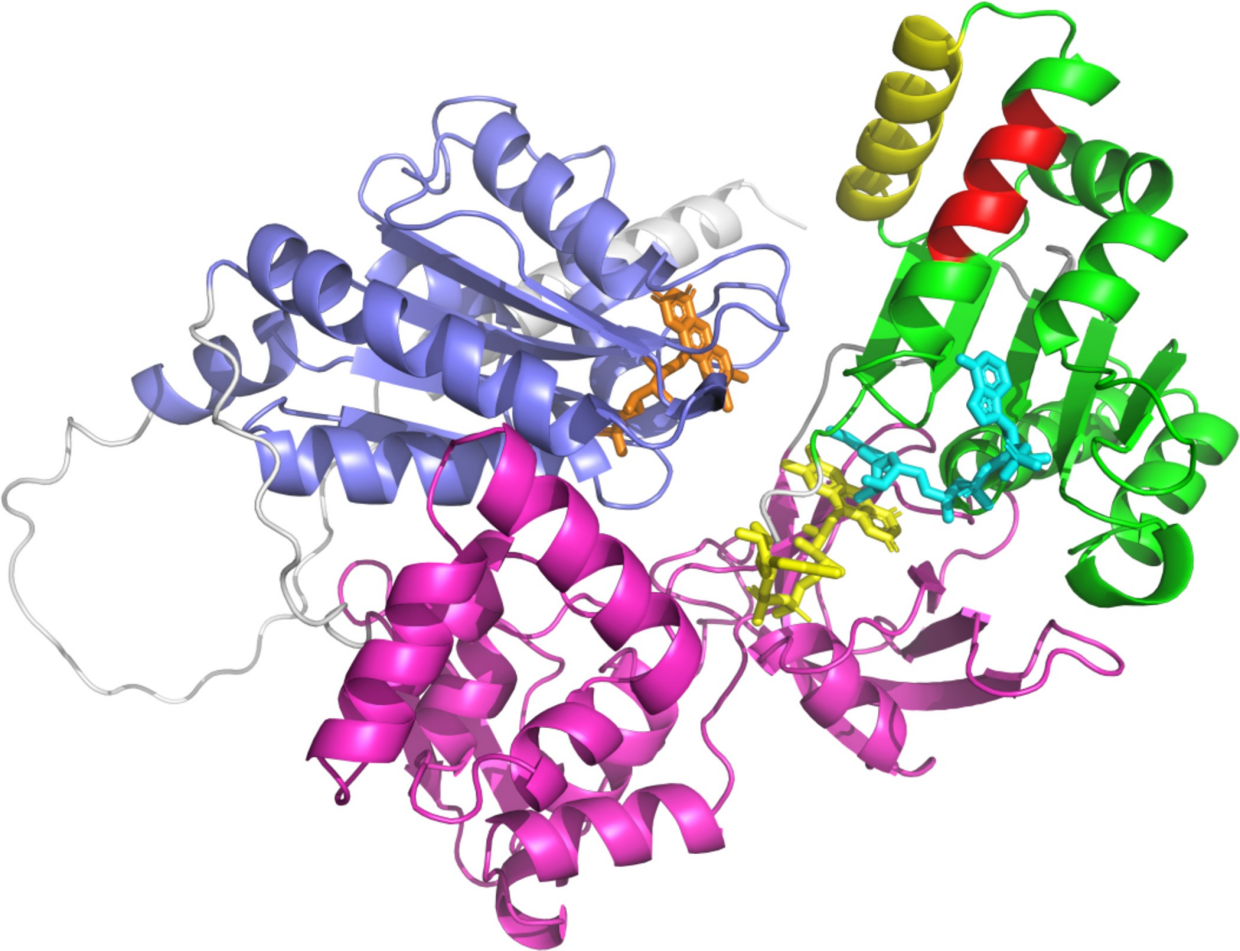
AlphaFold model of *Ld*P450R1 in closed conformation. The FMN, FAD and NADPH binding domains are highlighted in blue, magenta and green, respectively. The N-terminal membrane attachment domain is highlighted in grey. FMN (orange), FAD (yellow) and NADPH (cyan) are shown in stick representation with binding modes modelled from the rat P450R structure (1J9Z.pdb). Helix 21 (H21), known to directly interact with partner CYPs is highlighted in yellow. Amino acids 605–612, deleted in our AmB R1, form part of helix 20 (H20). Deleted amino acids are highlighted in red.

The structures of several P450R enzymes from a variety of species have been solved [45–48], with all sharing relatively similar architecture, comprised of FMN-, FAD- and NADPH-binding domains, as well as an *N*-terminal membrane anchor. The predicted AlphaFold structure of *Ld*P450R1 shares the same broad structure (**Fig 6**) [49,50]. Crucially, the model is predicted with high to very high confidence, and the catalytic residues overlay with those of the human enzyme [45]. Following binding of NADPH, electrons are transferred sequentially to FAD and then FMN, which are closely aligned for electron transfer while the enzyme is in its closed conformation. Electron transfer from NADPH to FMN induces a significant conformational change, ultimately driving the enzyme into an open conformation and creating the substrate (CYP) binding site [51]. The predominant P450R-CYP interaction is via the P450R FMN-binding domain, however, molecular dynamic simulations indicate that final helix of the NADPH-binding domain (helix 21) also interacts directly with CYP [52]. Amino acids 605–612, deleted in AmB R1, are located on helix 20 which directly interacts with the CYP-binding helix 21, as well as helices 18 and 19 which form the NADPH biding site. We hypothesise that deletion of residues 605–612 and the resulting truncation of helix 20 will likely disrupt key interactions with neighbouring helices, thus impacting NADPH and/or CYP51 binding.

## Conclusions

At the outset of these studies our principal goal was to add to the current understanding of AmB resistance mechanisms in *L*. *donovani*. In keeping with previous studies in *L*. *mexicana*, we confirmed that deletion of *SMT1* was the primary driver of the moderate resistance demonstrated by three out of four of our cell lines. Comprehensive genetic and biochemical analysis of our remaining hyper-resistant clone confirmed that these parasites were deficient in P450R1, a *bona fide* P450 reductase likely involved in catalytic regeneration of CYP51. These studies contribute to our general understanding of the *L*. *donovani* sterol biosynthetic pathway and pave the way for further investigations to understand the specific functions of the two remaining and uncharacterised *L*. *donovani* P450Rs. The fact that P450R1 is apparently essential for amastigote viability suggests that functional loss of this reductase is unlikely to be a major cause of AmB clinical resistance. However, as an essential enzyme in a pathway that is already the focus of anti-trypanosomal drug discovery efforts, P450R1 may represent an interesting prospect for future chemotherapeutic intervention.

## Materials and methods

### Cell lines and culture conditions

Clonal *Leishmania donovani Ld*BOB (derived from MHOM/SD/62/1SCL2D) was grown either as promastigotes or axenic amastigotes in media specific for each developmental stage [33]. Promastigotes were grown at 28°C while axenic amastigotes were grown at 37°C in 5% CO_2_.

#### Drug sensitivity assays

Drug sensitivity assays were carried out as previously described [53]. Data were processed using GRAFIT (version 5.0.4, Erithacus Software) and fitted to the 2-parameter equation shown below to determine the effective concentration inhibiting growth by 50% (EC_50_), where *[I]* is the inhibitor concentration, and *m* is the slope factor. Experiments were performed in biological replicate (>2) with the data presented as the weighted mean ± standard deviation.


$$y=\frac{100}{1+{\left(\frac{\left[I\right]}{{EC}_{50}}\right)}^{m}}$$

Biphasic dose response curves were fitted to the following equation, where A% is the amplitude of the low EC_50_ value:

$$y=\frac{A\%}{1+{\left(\frac{\left[I\right]}{{EC}_{50Low}}\right)}^{mLow}}+\frac{100-A\%}{1+{\left(\frac{\left[I\right]}{{EC}_{50High}}\right)}^{mHigh}}$$

### Generation of amphotericin B-resistant clones

Amphotericin B-resistant clones were generated through continuous culture of a promastigote *L*. *donovani* wild-type, drug-sensitive clone in the presence of increasing concentrations of amphotericin B. Starting at 20 nM (equivalent to 1× EC_50_), resistance was generated in 4 independent cultures, as previously described [53]. Once cultures were able to grow in concentrations of drug equivalent to 20× EC_50_, parasites were cloned by limiting dilution, and a single clone from each culture was selected for further investigation.

### Whole genome sequencing and analysis

Genomic DNA was isolated from WT and resistant clones via classical SDS-proteinase K-phenol-chloroform extraction. Whole genomic sequencing was performed using a DNBseq next-generation sequencing platform (Beijing Genomics Institute, Hong Kong). Sequencing reads, each 120 base pairs in length, were processed through the OVarFlow pipeline (release: May10_2021_BQSR) [54]. This pipeline was deployed within a Docker container and executed on a high-performance computing (HPC) cluster. Variant calling was performed against the LdBPK genome version 39 sourced from TriTrypDB [55] and augmented with the maxi-circle sequence obtained from GenBank (Accession No. CP022652.1). Additionally, the BAM files generated were utilised for variant calling via bcftools (formerly samtools [56]) (v 1.9).

Variant call format (VCF) files produced from these analyses underwent filtration using a custom Python script, which was designed to exclude variants uniformly called across all samples. This step was implemented to ensure the identification of unique variants specific to resistant clones as compared to the WT. Microsoft Excel was then utilised for the visualization and inspection of these VCF files, facilitating the identification of mutations. Mutation acceptance criteria included a genotype quality score of at least 99, a minimum of 20 supporting genotype reads (DP value 20) and an overall quality score of no less than 1000.

Gene copy number variations were assessed based on their Reads Per Kilobase of transcript, per Million mapped reads (RPKM) values. The bam files produced by the OVarFlow pipeline were used to compute the RPKM values. RPKM values were calculated utilising the rpkm function provided by the edgeR package (3.28.0) [57] in R, leveraging gene count data obtained through featureCounts (1.6.4) [58]. Chromosome copy number variation was visualised using the RPKM gene values.

For each WGS sample, the number of 120-bp reads containing the *SMT1*-specific subsequence 5´-GCACGTACAAGGCGACGGAGGTTTTGGAGGAGGCTGCGGAA-3´ and *SMT2*-specific subsequence 5´-GCACGTACAAGGCGACGGAGATTTTGGAGGAGGCTGCGGAA-3´ were counted using a custom script implemented in Python and expressed as percentage of total SMT-specific reads. SMT1 and SMT2 RPKM values were adjusted by multiplying the total SMT RPKM by percentage SMT1 and SMT2. True SMT1 and SMT2 gene copy numbers were calculated by dividing their adjusted RPKM by the average RPKM for chromosome 36 and multiplying by chromosome 36 ploidy.

All WGS datasets have been deposited with the National Centre for Biotechnology Information Sequence Read Archive (NCBI SRA) under project code PRJNA994719. The code used in this project has been deposited in GitHub (https://github.com/mtinti/amphotericin_Ldonovani) and archived in Zenodo (https://zenodo.org/records/10567623).

### Expression of SMT1 and P450R1 in AmB-resistant cell lines

The genes encoding sterol C24-methyltransferase 1 (*SMT1*, LdLV9.36.2.209980) and 2 (*SMT2*, LdLV9.36.2.209990) were PCR-amplified from *L*. *donovani* wild-type genomic DNA using primers LBT-032 and LBT-033 (**S5 Table**) and Q5 polymerase (NEB), as per manufacturers’ instructions. The full-length genes were inserted into the overexpression plasmid pIR1SAT via a *BglII* site. Similarly, the gene encoding the putative P450 reductase (*P450R1*, LdBPK_281350.1) was amplified from genomic DNA harvested from wild-type and AmB R3 parasites using primers LBT-150 and LBT-151. The resulting wild-type and mutated genes were cloned into pIR1SAT via *Xma*I and *Xba*I restriction sites. Mid-log AmB R3, *SMT1/2* DKO, AmB R1 or *P450R1* DKO promastigotes (10^7^) were transfected with 10 μg of pIR1-SMT1 or pIR1-P450R, respectively, as previously described [59]. The resulting cultures were selected with 100 μg/ml nourseothricin, and clones were isolated by limiting dilution.

### Introduction of P450R INDEL in WT promastigotes

Nucleotides 1813–1836 of *P450R* (P450R Δ605–612) were deleted from *Ld*BOB WT parasites constitutively expressing Cas9 and T7 RNA polymerase yielding the P450R1^Δ605–612^ cell line, as previously described [60,61]. Briefly, the single guide RNA (sgRNA) directing Cas9 to nucleotide 1834 was generated through PCR-extension of primer LBT-153 with primer G00, using the protocol established by Gluenz and colleagues [61]. The resulting sgRNA alongside the accompanying repair template (LBT-152) were transfected into promastigotes simultaneously, as described [59].

### Generation of gene knockouts

Gene knockouts were engineered using CRISPR-Cas9. Briefly, two sgRNAs were generated targeting the 5´ and 3´ regions of the target genes (*SMT1*, *SMT2* and *P450R1*). Repair templates comprised of a resistance cassette flanked by 25 nucleotides homologous to the 5’- and 3’-UTR regions of target genes. All primers used for the generation of sgRNA and repair templates were designed using LeishGEdit ([62], http://www.leishgedit.net/Home.html).

Specifically, for the generation of *SMT1* SKO, sgRNA templates directing Cas9 cleavage 5´ and 3´ to the *SMT1* gene were generated through PCR-extension of primers LBT-036 and LBT-037 respectively with primer G00. A puromycin KO cassette repair template was generated through PCR-amplification of pTPuro_v1 [61] with LBT-034 and LBT-035. Transfected cells were selected with 20 μg/ml puromycin, and clonal parasites were generated by limiting dilution. *SMT1* DKO parasites were generated by repeating this process with confirmed SKO cells, using the same sgRNA templates. However, in this instance the repair template was generated through PCR-amplification of pTBlast_v1 [61] with primers LBT-034 and LBT-035. Transfected SKO promastigotes were selected with 20 μg/ml puromycin and 10 μg/ml blasticidin, and clonal DKO parasites were isolated by limiting dilution.

All other DKO lines described were generated following a single round of transfection. For SMT2 (LdLV9.36.2.209990), sgRNA templates were generated through PCR-extension of primers LBT-040 and LBT-041 respectively with primer G00, and the repair templates were generated through PCR-amplification of pTPuro_v1 and pTBlast_v1 with primers LBT-038 and LBT-039. For SMT1 and 2 dual KO, sgRNA templates were generated through PCR-extension of primers LBT-036 and LBT-041 respectively with primer G00, and the repair templates were generated through PCR-amplification of pTPuro_v1 and pTBlast_v1 with primers LBT-034 and LBT-039. For P450R1 (LdBPK_281350.1), sgRNA templates were generated through PCR-extension of primers LBT-156 and LBT-157 respectively with primer G00, and the repair templates were generated through PCR-amplification of pTPuro_v1 and pTBlast_v1 with primers LBT-154 and LBT-155. For CYP51 (LdBPK_111100.1), sgRNA templates were generated through PCR-extension of primers LBT-169 and LBT-170 respectively with primer G00, and the repair templates were generated through PCR-amplification of pTPuro_v1 and pTBlast_v1 with primers LBT-167 and LBT-168.

For each DKO cell line, relevant sgRNA and repair templates were combined and transfected into WT *L*. *donovani* promastigotes constitutively expressing Cas9 and T7 RNA polymerase, as described above. Transfected cells were selected with 20 μg/ml puromycin and 10 μg/ml blasticidin 24 h following transfection. Clonal parasites were generated for each line by limiting dilution and confirmed as null for our genes of interest by whole genome sequencing.

### Quantitative RT-PCR

RNA was harvested from 10^8^ promastigotes using the RNeasy kit (Qiagen) according to the manufacturer’s instructions. Residual DNA was digested from samples with RNase-Free DNase (Qiagen). Quantitative RT-PCR was performed with 100 ng of total RNA using a Luna Universal One-Step RT-qPCR kit (New England Biolabs) as previously described [63]. Relative quantification was established using the established reference gene *rRNA45* [64]. Primers were designed using the Primer3Plus website. For total *SMT* RNA quantitation, primers LBT-090 and LBT-091 were used, and for P450R RNA quantitation primers LBT-177 and LBT-178 were used. The levels of each RNA transcript in AmB-resistant and transgenic clones were normalised to WT using the ΔΔCT method. Two biological replicates were performed for each analysis.

### Quantitative proteomics

#### Sample preparation

*L*. *donovani* cell lysates were prepared precisely as previously described [53]. Protein digests were processed using S-Trap (Protifi) according to the manufacturer’s recommendations. Briefly, 50 μg protein was solubilised in 5% SDS, reduced with 10 mM tris(2-carboxyethyl)phosphine (TCEP) for 15 min at 55°C, alkylated with 40 mM iodoacetamide for 30 min at RT in the dark. Alkylated proteins were suspended in the presence of 2.5% H_3_PO_4_, captured on a S-Trap micro column where they were washed and then digested with Trypsin/LysC at 10:1 protein:enzyme ratio at 37°C for ~16h. Peptides were eluted with a combination of aqueous and organic buffers and dried on a vacuum evaporator.

#### Mass spectrometry analysis

LC-MS/MS analysis was performed by the FingerPrints Proteomics Facility (University of Dundee) on a Orbitrap Astral mass spectrometer (Thermo Scientific) coupled with a Vanquish Neo HPLC (Thermo Scientific). LC buffers used were as follows: Buffer A (0.1% formic acid in Milli-Q water (v/v)) and Buffer B (80% acetonitrile and 0.1% formic acid in Milli-Q water. Aliquots (15 μl) were loaded at 60 μl/min onto a trap column (PepMap Neo C18 5 μm 300 μm x 5 mm, Thermo Scientific) pre-equilibrated with 96% Buffer A. The trap column was washed for 5 min at 200 μl/min and then the trap column was switched in-line with a Thermo Scientific, resolving column (PepMap RSLC C18, 2 μm, 150 μm x 15 cm). The peptides were eluted from the column at a constant flow rate of 1.3 μl/min with a gradient from 4% buffer to 22.5% Buffer B for 13.9 min, 35% B for 6.9 min, 55% B for 0.5 min and then 99% Buffer B by 21.7 min. The column was then washed with 99% Buffer B for 0.9 min and re-equilibrated in 4% Buffer B. The Orbitrap Astral was operated in positive mode using data-independent mode. A scan cycle comprised an Orbitrap MS1 scan (m/z range from 380–980, with a maximum ion injection time of 5 ms, a resolution of 240,000 and automatic gain control (AGC) value of 500% followed by 149 Astral DIA scans (with an isolation window set to 4 m/z, maximum ion injection time at 3 ms and AGC 500%). HCD collision energy was set to 25. To ensure mass accuracy, the mass spectrometer was calibrated on day one of analysis.

#### Data analysis

Protein search was performed in DIA-NN (version 1.8.1) using a library-free search. An *in-silico* library was generated using the *L*. *donovani* BPK282A1 proteome (UP000008980 from Uniprot.org). Searches included cabamidomethylation as a fixed modification and acetylation (*N*-terminus) and oxidation (methionine) as variable modifications. The match between runs option was active. All proteomics datasets have been deposited to the ProteomeXchange Consortium via the PRIDE partner repository under the identifier **PXD052472.**

### Macrophage infectivity assays

In-macrophage infectivity assays were carried out using starch-elicited mouse peritoneal macrophages harvested from BALB/c mice and metacyclic promastigotes, as previously described [65].

### Sterol profiling

Sterols were extracted from mid-log promastigotes (3 × 10^8^ per sample) and analysed via GC-MS, as previously described [25]. Sterol-associated peaks in GC-MS data were assigned through direct matches to authentic sterol standards or through retention times and/or ion patterns associated with previously identified sterols [25] (details of each peak assignment summarised in **S6 Table**). Sterols were then mapped to the *Leishmania* ergosterol biosynthesis pathway proposed by Zhang and co-workers ([41], **Fig 1**). Analysis was carried out on two biological replicates.

## Supporting information

S1 Table SNPs, gene deletions and INDELs identified in AmB-resistant clones. *Identified through homology to the *L*. *donovani* LV9 genome due to poor annotation of the *L. donovani* LdBPK genome at this locus.
(DOCX)

S2 Table Copy number variations in AmB-resistant cell lines. Increased chromosomal copy number versus WT highlighted in green and reduced copy highlighted in red.
(DOCX)

S3 Table Analysis of the *SMT* locus in WT, AmB-resistant and transgenic cell lines via whole genome sequencing. Strategy to distinguish reads associated with *SMT1* and *2* outlined in Materials and Methods.
(DOCX)

S4 Table Collated AmB EC_50_ values for WT, resistant and transgenic axenic amastigote cell lines. NS–non-surviving—parasites did not survive differentiation to axenic amastigotes. EC_50_ values represent the weighted mean ± standard deviation of indicated number of biological replicates with each biological replicate comprised of at least two technical replicates.
(DOCX)

S5 Table List of primers used in this study. (DOCX)

S6 Table Identification of sterol-associated peaks in GC-MS data. RT – retention time; FF-MAS - Follicular fluid meiosis-activating sterol; T-MAS - 4,4-dimethyl cholest-8(9),24-dien-3β-ol. *All masses reported as monoisotopic mass.
(DOCX)

S7 Table Sterol composition of WT, AmB-resistant and transgenic *L*. *donovani* promastigotes. Values are the mean of biological replicates and represent % of total. The substrates of CYP51 are highlighted in bold. See Fig 1 for pathway details. ND–not detected.
(DOCX)

S8 Table Collated ketoconazole EC_50_ values for WT, resistant and transgenic cell lines. EC_50_ values represent the weighted mean ± standard deviation of three biological replicates with each biological replicate comprised of two technical replicates.
(DOCX)

S1 Fig Relative *SMT* RNA transcript levels in AmB-resistant and transgenic promastigote cell lines. Data represent the mean ± SD of triplicate determinations.
(TIF)

S2 Fig Relative *SMT* RNA transcript levels in WT and transgenic axenic amastigote cell lines. Data represent the mean ± SD of triplicate determinations.
(TIF)

S3 Fig Relative P450R1 and SMT1/2 protein levels in selected WT, resistant and transgenic cell lines. Protein levels (relative to WT) were determined by label free quantitation. SMT1/2 relative expression in promastigotes (A) and axenic amastigotes (B). (C) P450R1 protein levels (relative to WT) in promastigotes. Details of these analyses can be found in the Materials and Methods.
(TIF)

S4 Fig Assessing the impact of *SMT1* and *SMT2* addback on AmB susceptibility. Dose-response curves for WT (white), *SMT1/2* DKO (blue), *SMT1/2* DKO plus *SMT1*^*WT*^ add-back (green) and *SMT1/2* DKO plus *SMT2*^*WT*^ add-back (red) promastigotes treated with AmB. EC_50_ values of 22 ± 0.1, 191 ± 2, 16 ± 0.01, and 18 ± 0.7 nM were determined for WT, *SMT1/2* DKO, *SMT1/2* DKO plus *SMT1*^WT^ and *SMT1/2* DKO plus *SMT2*^WT^ promastigotes, respectively. These EC_50_ curves and values represent one biological replicate, composed of two technical replicates. Collated datasets reporting the weighted mean ± SD of multiple biological replicates are summarised in **Table 1**.
(TIF)

S5 Fig Assessing the impact of modulating CYP51 and P450R1 levels on infectivity. Mean numbers of WT, DKO and resistant amastigotes infecting mouse peritoneal macrophages were determined following 72 h incubations. Bar represents the mean value of two biological replicates with the individual data points also shown.
(TIF)
