# Supplementary material for: Sterol 14-alpha demethylase (CYP51) activity in Leishmania donovani is likely dependent upon cytochrome P450 reductase 1
Source: PLoS Pathog. 2024 Jul 11;20(7):e1012382. doi: 10.1371/journal.ppat.1012382 (PMC11265716; doi:10.1371/journal.ppat.1012382)
Supplement: S1 Table — *Identified through homology to the L. donovani LV9 genome due to poor annotation of the L. donovani LdBPK genome at this locus. (DOCX) [file ppat.1012382.s001.docx]

| **Gene ID** | **Function** | **Mutation** | | **Cell line** |
| --- | --- | --- | --- | --- |
| LdBPK_041200.1 | Hypothetical protein | 3-bp insertion | Heterozygous | R1, R2, R4 |
|  |  | 37-bp insertion (frameshift) | Heterozygous | R1, R2, R4 |
| LdBPK_070700.1 | Vacuolar-type Ca^2+^-ATPase, putative (fragment) | 7-bp deletion (frameshift) | Heterozygous | R2 |
| **LdBPK_201210.1** | **Calpain-like cysteine peptidase, putative** | **26-bp insertion (frameshift)** | **Homozygous** | **R4** |
| LdBPK_210920.1 | Plectin, putative | 58-bp insertion (frameshift) | Heterozygous | R2, R3 |
|  |  | 1-bp deletion (frameshift) | Heterozygous | R2, R3 |
| LdBPK_211100.1 | Mis-match repair protein, putative | SNP (P153L) | Heterozygous | R3 |
| LdBPK_221010.1 | ChaC-like protein, putative | 2-bp deletion with frameshift | Heterozygous | R2, R3, R4 |
| LdBPK_240620.1 | SWI/SNF-related matrix-associated actin-dependent regulator of chromatin subfamily A-like protein, putative | SNP (A123T) | Heterozygous | R2 |
| LdBPK_262350.1 | 60S ribosomal protein L35, putative | 75-bp insertion | Heterozygous | R1, R3, R4 |
| **LdBPK_281350.1** | **P450 reductase, putative** | **Deletion (Δ605-612)** | **Homozygous** | **R1** |
| LdBPK_312290.1 | Hypothetical protein | 16-bp deletion (frameshift) | Heterozygous | R1, R2 |
|  |  | 10-bp deletion (frameshift) | Heterozygous | R1 |
| LdBPK_333360.1 | β'-COP protein | SNP (D516Y) | Heterozygous | R3 |
|  |  | SNP (D516E) | Heterozygous | R3 |
| LdBPK_360990.1 | 40S ribosomal protein S18, putative | 69-bp insertion | Heterozygous | R1 |
| **LdLV9.36.2.209980*** | **Sterol C24-methyltransferase 1 (SMT1)** | **Frameshift (premature STOP)** | **Homozygous** | **R3** |
|  |  | **Gene deletion** | **Homozygous** | **R2, R4** |
| LdBPK_365480.1 | Hypothetical protein | 1-bp insertion (frameshift) | Heterozygous | R2 |
| LdBPK_365480.1 |  | 52-bp insertion (frameshift) | Heterozygous | R2 |
| LdBPK_366560.1 | Glucose transporter 1 | 18-bp insertion | Heterozygous | R1, R2, R3 |
