# Supplementary material for: Sterol 14-alpha demethylase (CYP51) activity in Leishmania donovani is likely dependent upon cytochrome P450 reductase 1
Source: PLoS Pathog. 2024 Jul 11;20(7):e1012382. doi: 10.1371/journal.ppat.1012382 (PMC11265716; doi:10.1371/journal.ppat.1012382)
Supplement: S2 Table — Increased chromosomal copy number versus WT highlighted in green and reduced copy highlighted in red. (DOCX) [file ppat.1012382.s002.docx]

| **Chromosome** | **Chromosome copy number** | | | | | |
| --- | --- | --- | --- | --- | --- | --- |
|  | **WT** | **AmB-R1** | **AmB-R2** | **AmB-R3** | **AmB-R4** |  |
| 1 | 2 | 2 | 2 | 2 | 2 |  |
| 2 | 2 | 2 | 2 | 2 | 2 |  |
| 3 | 2 | 2 | 3 | 2 | 2 |  |
| 4 | 2 | 2 | 2 | 2 | 2 |  |
| 5 | 2 | 2 | 2 | 2 | 2 |  |
| 6 | 2 | 2 | 2 | 2 | 2 |  |
| 7 | 2 | 2 | 2 | 2 | 2 |  |
| 8 | 2 | 2 | 3 | 2 | 2 |  |
| 9 | 2 | 2 | 2 | 2 | 2 |  |
| 10 | 2 | 2 | 2 | 2 | 2 |  |
| 11 | 2 | 2 | 2 | 2 | 2 |  |
| 12 | 2 | 2 | 2 | 2 | 2 |  |
| 13 | 2 | 2 | 2 | 2 | 2 |  |
| 14 | 2 | 2 | 2 | 2 | 2 |  |
| 15 | 2 | 2 | 2 | 2 | 2 |  |
| 16 | 2 | 2 | 3 | 2 | 2 |  |
| 17 | 2 | 2 | 2 | 2 | 2 |  |
| 18 | 2 | 2 | 2 | 2 | 2 |  |
| 19 | 2 | 2 | 4 | 2 | 2 |  |
| 20 | 2 | 2 | 2 | 2 | 2 |  |
| 21 | 2 | 2 | 4 | 2 | 2 |  |
| 22 | 4 | 3 | 4 | 4 | 4 |  |
| 23 | 2 | 2 | 3 | 2 | 2 |  |
| 24 | 2 | 2 | 2 | 2 | 2 |  |
| 25 | 2 | 2 | 2 | 2 | 2 |  |
| 26 | 2 | 2 | 3 | 3 | 3 |  |
| 27 | 2 | 2 | 2 | 2 | 2 |  |
| 28 | 2 | 2 | 2 | 2 | 2 |  |
| 29 | 2 | 2 | 3 | 2 | 2 |  |
| 30 | 2 | 2 | 2 | 2 | 2 |  |
| 31 | 4 | 4 | 5 | 4 | 4 |  |
| 32 | 2 | 2 | 2 | 2 | 2 |  |
| 33 | 2 | 2 | 4 | 2 | 2 |  |
| 34 | 2 | 2 | 3 | 2 | 2 |  |
| 35 | 2 | 2 | 2 | 2 | 2 |  |
| 36 | 2 | 2 | 2 | 2 | 2 |  |
