# Supplementary material for: Sterol 14-alpha demethylase (CYP51) activity in Leishmania donovani is likely dependent upon cytochrome P450 reductase 1
Source: PLoS Pathog. 2024 Jul 11;20(7):e1012382. doi: 10.1371/journal.ppat.1012382 (PMC11265716; doi:10.1371/journal.ppat.1012382)
Supplement: S3 Table — Strategy to distinguish reads associated with SMT1 and 2 outlined in Materials and Methods. (DOCX) [file ppat.1012382.s003.docx]

| **Cell line** | **Read counts** | | **RPKM** | | **Gene copy number** | |
| --- | --- | --- | --- | --- | --- | --- |
|  | **SMT1** | **SMT2** | **Ch. 36** | **Total SMT** | **SMT1** | **SMT2** |
| WT | 42 | 41 | 28 | 58 | 2 | 2 |
| R1 | 34 | 40 | 28 | 57 | 2 | 2 |
| R2 | 0 | 33 | 22 | 21 | 0 | 2 |
| R3 | 47 | 52 | 27 | 53 | 2 | 2 |
| R4 | 0 | 34 | 27 | 26 | 0 | 2 |
| *SMT1* DKO | 0 | 64 | 59 | 59 | 0 | 2 |
| *SMT2* DKO | 58 | 0 | 60 | 57 | 2 | 0 |
| *SMT1/2* DKO | 1 | 0 | 60 | 0 | 0 | 0 |
