# Supplementary material for: Sterol 14-alpha demethylase (CYP51) activity in Leishmania donovani is likely dependent upon cytochrome P450 reductase 1
Source: PLoS Pathog. 2024 Jul 11;20(7):e1012382. doi: 10.1371/journal.ppat.1012382 (PMC11265716; doi:10.1371/journal.ppat.1012382)
Supplement: S4 Table — NS–non-surviving—parasites did not survive differentiation to axenic amastigotes. EC50 values represent the weighted mean ± standard deviation of indicated number of biological replicates with each biological replicate comprised of at least two technical replicates. (DOCX) [file ppat.1012382.s004.docx]

| **Cell line** | **^*^EC_50_ value, nM** | **Fold shift (relative to WT)** | **Biological replicates** |
| --- | --- | --- | --- |
| WT | 137 ± 6 | - | 7 |
| AmB R1 | NS | - | - |
| AmB R1 + P450R1^WT^ | 121 ± 8 | 1 | 3 |
| AmB R1 + P450R1 ^Δ605-612^ | NS | - | - |
| AmB R3 | 671 ± 34 | 5 | 5 |
| AmB R3 + SMT1^WT^ | 145 ± 14 | 1 | 3 |
| AmB R3 + SMT2^WT^ | 127 ± 7 | 1 | 3 |
| SMT1 DKO | 573 ± 50 | 4 | 3 |
| SMT2 DKO | 90 ± 10 | 1 | 2 |
| SMT1/2 DKO | 934 ± 71 | 7 | 6 |
| SMT1/2 DKO + SMT1^WT^ | 79 ± 7 | 1 | 3 |
| SMT1/2 DKO + SMT2^WT^ | 94 ± 7 | 1 | 3 |
| P450R1^Δ605-612^ | NS | - | - |
| P450R1 DKO | NS | - | - |
| P450R1 DKO + P450R1^WT^ | 264 ± 26 | 2 | 3 |
| P450R1 DKO + P450R1^Δ605-612^ | NS | - | - |
| CYP51 DKO | 10300 ± 1600 | 75 | 3 |
|  | | |  |
