# Supplementary material for: Sterol 14-alpha demethylase (CYP51) activity in Leishmania donovani is likely dependent upon cytochrome P450 reductase 1
Source: PLoS Pathog. 2024 Jul 11;20(7):e1012382. doi: 10.1371/journal.ppat.1012382 (PMC11265716; doi:10.1371/journal.ppat.1012382)
Supplement: S5 Table — (DOCX) [file ppat.1012382.s005.docx]

| **Primer** | **Sequence (5´– 3´)** |
| --- | --- |
| LBT 032 | TTAATAAGATCTATGTCCGCCGGTGGCCGTGAGACCGC |
| LBT 033 | TTAATAAGATCTCTAAGCCTGCTTGGACGGCTTGCGAGC |
| LBT 030 | TTAATAAGATCTaacctccgcggtcctctgaagatctctagg |
| LBT 031 | TTAATAAGATCTacttttgcaaagcaagaaaaaagtcc |
| LBT 058 | CCTGGTGACGGACTTCTACG |
| LBT 059 | CTCGATGCGGTGCTTGATTG |
| LBT 064 | GAGTCCGCAGCCGTAGCCGAGGG |
| LBT 065 | ccgtcgctgctccacacacagcc |
| LBT 150 | TAATAACCCGGGATGTCTTTCCTCTACATTGTCGCC |
| LBT 151 | TAATAATCTAGACTACAAGGTCGATGACCACACATCCTTTAGG |
| LBT 171 | AATAATAGATCTATGATCGGCGAGCTACTCCTTCTCC |
| LBT 172 | AATAATAGATCTCTAAGCAGCCGCCTTCTTCTTCTTGATG |
| LBT 030 | TTAATAAGATCTaacctccgcggtcctctgaagatctctagg |
| LBT 031 | TTAATAAGATCTacttttgcaaagcaagaaaaaagtcc |
| LBT 032 | TTAATAAGATCTATGTCCGCCGGTGGCCGTGAGACCGC |
| LBT 033 | TTAATAAGATCTCTAAGCCTGCTTGGACGGCTTGCGAGC |
| LBT 034 | TGTACACTACCTCTTCGCTTGTTTTTTCCCgtataatgcagacctgctgc |
| LBT 035 | ctacagtgtgggcactctaccccgataccaccaatttgagagacctgtgc |
| LBT 036 | gaaattaatacgactcactataggTGTAGGTGTGGGGGTAAGTAgttttagagctagaaatagc |
| LBT 037 | gaaattaatacgactcactataggtgatgccagccacccgaccggttttagagctagaaatagc |
| LBT 038 | ccatctacaaggtcatttctttcgtcaaccgtataatgcagacctgctgc |
| LBT 039 | acgaacttttgcaaagcaagaaaaaagtccccaatttgagagacctgtgc |
| LBT 040 | gaaattaatacgactcactataggcgtgtaggtgtgaggctgtggttttagagctagaaatagc |
| LBT 041 | gaaattaatacgactcactataggtgtttttgtttcaagagccagttttagagctagaaatagc |
| LBT 064 | GAGTCCGCAGCCGTAGCCGAGGG |
| LBT 065 | ccgtcgctgctccacacacagcc |
| LBT 090 | CGACTTCTGCAACATGAGCTT |
| LBT 091 | TACGTGCCCTTCGGAGCTA |
| LBT 150 | TAATAACCCGGGATGTCTTTCCTCTACATTGTCGCC |
| LBT 151 | TAATAATCTAGACTACAAGGTCGATGACCACACATCCTTTAGG |
| LBT 152 | ACGCGAGGCGCATGGCCAAGGACGTGGAGGTGGAGGGCAAGATGCCGCGC |
| LBT 153 | gaaattaatacgactcactataggGGCGGAGCTCAAGCGCATTGgttttagagctagaaatagc |
| LBT 154 | ATCTGGCCTCGTCGGCTAGTCCCCAACCCCgtataatgcagacctgctgc |
| LBT 155 | GCTTCATGATGTTCTCGCCTTTCTGTACTTccaatttgagagacctgtgc |
| LBT 156 | gaaattaatacgactcactataggGCTACAGTCGAACGAGGGAGgttttagagctagaaatagc |
| LBT 157 | gaaattaatacgactcactataggCTCTCTTTCCCGCTCCACCGgttttagagctagaaatagc |
| LBT 163 | GGCAAGAGAGAGGCAGCGACGACACTCCC |
| LBT 164 | GCCTCCTCATACGGAGACGGTCGCTCCC |
| LBT 167 | CTCCCCCTTTCGCTGTCGCTCGTACCACCGgtataatgcagacctgctgc |
| LBT 168 | CACACGCACGTGCTCTCAGCGCCGTCTCCGccaatttgagagacctgtgc |
| LBT 169 | gaaattaatacgactcactataggTGCTCTTTCGATGTAGTGTGgttttagagctagaaatagc |
| LBT 170 | gaaattaatacgactcactataggCGATGAAAACCCGAGAGAGAgttttagagctagaaatagc |
| LBT 171 | aataatagatctATGATCGGCGAGCTACTCCTTCTCC |
| LBT 172 | aataatagatctCTAAGCAGCCGCCTTCTTCTTCTTGATG |
| LBT 173 | CTCTTTGCTTGCGCTTGCCGTTGCC |
| LBT 174 | AGAGAAGCGACAACAACAAACGGCG |
| LBT 177 | CCATCATCCGCAACGAAGAG |
| LBT 178 | CCGGCTTGGTAGGAGATGAT |
