# Supplementary material for: Sterol 14-alpha demethylase (CYP51) activity in Leishmania donovani is likely dependent upon cytochrome P450 reductase 1
Source: PLoS Pathog. 2024 Jul 11;20(7):e1012382. doi: 10.1371/journal.ppat.1012382 (PMC11265716; doi:10.1371/journal.ppat.1012382)
Supplement: S6 Table — RT – retention time; FF-MAS - Follicular fluid meiosis-activating sterol; T-MAS - 4,4-dimethyl cholest-8(9),24-dien-3β-ol. *All masses reported as monoisotopic mass. (DOCX) [file ppat.1012382.s006.docx]

|  |  | **Sterol** | **Major** | **Molecular** | |  |  |
| --- | --- | --- | --- | --- | --- | --- | --- |
|  |  | **Type** | **Fragment*** | **Ion* (*m/z*)** | | **RT** |  |
| **Sterol** | **Formula** | **(C:DB)** | **(*m/z*)** | **+TMS** | **-TMS** | **(min)** | **Basis of match** |
| Cholesterol | C27H46O | 27:1 | 368 | 458 | 386 | 10.40 | Match to standard |
| Desmosterol | C27H44O | 27:2 | 456 | 456 | 384 | 10.63 | Match to standard |
| 14-Methylzymosterol | C28H46O | 28:2 | 365 | 470 | 398 | 10.73 | Matches predicted RT. Corresponds with sterol accumulated in CYP51-deficient *L. mexicana* [66] |
| Cholesta-7-enol | C27H46O | 27:1 | 441 | 458 | 386 | 10.77 | Match to standard |
| Zymosterol | C27H44O | 27:2 | 351 | 456 | 384 | 10.77 | Match to standard |
| 7-dehydrodesmosterol | C27H42O | 27:3 | 349 | 455 | 382 | 10.89 | Only 27:3;O sterol in the pathway. Previously dectected as principal sterol in SMT1-defecient *L. mexicana* promastigotes [25] |
| Ergosterol | C28H44O | 28:3 | 363 | 468 | 396 | 10.90 | Match to standard |
| Cholesta-7,24-dienol | C27H44O | 27:2 | 343 | 456 | 384 | 11.00 | Does not match the RT for desmosterol or zymosterol. Cholesta-7,24-dienol is only remaining C28-dien in the pathway |
| Ergosta-5,7,22,24-tetraenol | C28H42O | 28:4 | 361 | 466 | 394 | 11.04 | Only 28:4;O sterol in the pathway |
| 14-Methylfecosterol | C29H48O | 29:2 | 379 | 484 | 412 | 11.05 | Matches predicted RT. Corresponds with the most abundant sterol in CYP51-deficient *L. mexicana* [66] |
| Fecosterol | C28H46O | 28:2 | 366 | 470 | 397 | 11.12 | Elevated in SC5D-deficient *L. mexicana* [66] Not detected |
| Stigmasterol | C29H48O | 29:2 | 394 | 484 | 412 | 11.17 | Match to standard |
| 4,14-Dimethylzymosterol | C29H48O | 29:2 | 484 | 484 | 412 | 11.21 | Does not match the predicted RT of 14-methylfecosterol or T-MAS. |
| 5-dehydroepisterol | C28H44O | 28:3 | 363 | 468 | 396 | 11.23 | Match to standard |
| 4-Methylzymosterol | C28H46O | 28:2 |  |  | 398 | 11.27 | Predicted RT Not detected |
| Episterol | C28H46O | 28:2 | 343 | 470 | 398 | 11.34 | Principle sterol in SC5D-defective *L. mexicana* promastigotes [25] |
| Lanosterol | C30H50O | 30:2 | 393 | 498 | 426 | 11.53 | Match to standard |
| Sitosterol | C29H50O | 29:1 | 396 | 486 | 414 | 11.54 | Match to standard |
| T-MAS | C29H48O | 29:2 |  |  | 412 | 11.59 | Predicted RT. Known to elute after sitosterol Not detected |
| FF-MAS | C29H46O | 29:3 | 377 | 482 | 410 | 11.60 | Match to standard |
