# Supplementary material for: Sterol 14-alpha demethylase (CYP51) activity in Leishmania donovani is likely dependent upon cytochrome P450 reductase 1
Source: PLoS Pathog. 2024 Jul 11;20(7):e1012382. doi: 10.1371/journal.ppat.1012382 (PMC11265716; doi:10.1371/journal.ppat.1012382)
Supplement: S7 Table — Values are the mean of biological replicates and represent % of total. The substrates of CYP51 are highlighted in bold. See Fig 1 for pathway details. ND–not detected. (DOCX) [file ppat.1012382.s007.docx]

| **Sterol** | **WT** | **AmB-R1** | **AmB-R1 + *P450R1*^WT^** | **AmB-R1 + *P450R1*** **^Δ605-612^** | ***P450R1*^Δ605-612^** | ***P450R1* DKO** |
| --- | --- | --- | --- | --- | --- | --- |
| Cholesterol | 2.8 ± 0 | 3.2 ± 0.1 | 2.2 ± 0.4 | 2.4 ± 0.1 | 2.2 ± 0.1 | 2.4 ± 0.2 |
| Desmosterol | 0.3 ± 0.1 | ND | 0.3 ± 0.1 | ND | ND | ND |
| **14-Methylzymosterol** | **0.7 ± 0.1** | **9.9 ± 0.1** | **4.9 ± 0.1** | **11.1 ± 0.6** | **9.1 ± 0.5** | **12.3 ± 1.1** |
| Cholesta-7-enol | ND | ND | ND | ND | ND | ND |
| Zymosterol | 0.7 ± 0 | ND | 0.7 ± 0.1 | ND | ND | ND |
| Ergosterol | 17.8 ± 1.0 | 0.2 ± 0.1 | 13.3 ± 2.3 | 0.1 ± 0 | 0.1 ± 0.1 | 0.2 ± 0.1 |
| Cholesta-7,24-dienol | 12.4 ± 0.8 | ND | 8.2 ± 0.1 | ND | ND | ND |
| Ergosta-5,7,22,24-tetraen-3β-ol | 2.7 ± 0.2 | ND | 1.6 ± 0.1 | ND | ND | 0.2 ± 0.1 |
| **14-methylfecosterol** | **0.1 ± 0.1** | **75.1 ± 0.4** | **2.3 ± 0.1** | **77.2 ± 1.6** | **68.8 ± 1.4** | **77.5 ± 1.6** |
| Stigmasterol | ND | 0.3 ± 0.4 | ND | 0.2 ± 0.3 | 0.8 ± 0 | 0.2 ± 0.3 |
| **4,14-dimethylzymosterol** | **1.0 ± 0.4** | **5.5 ± 0.4** | **1.8 ± 0** | **5.1 ± 0.8** | **8.6 ± 1.2** | **4.1 ± 0.4** |
| 5-dehydroepisterol | 46.5 ± 0.9 | 0.8 ± 0.1 | 57.7 ± 2.8 | 0.6 ± 0.2 | 0.8 ± 0.3 | 0.7 ± 0.2 |
| Episterol | 13.5 ± 0.7 | ND | 6.1 ± 0.6 | ND | ND | ND |
| **Lanosterol** | **1.6 ± 0** | **5.2 ± 0.4** | **1.0 ± 0.1** | **3.2 ± 0.4** | **9.5 ± 0.1** | **2.6 ± 0.1** |
| Sitosterol | ND | ND | ND | ND | ND | ND |
| FF-MAS | ND | ND | ND | ND | ND | ND |
