# Supplementary material for: Sterol 14-alpha demethylase (CYP51) activity in Leishmania donovani is likely dependent upon cytochrome P450 reductase 1
Source: PLoS Pathog. 2024 Jul 11;20(7):e1012382. doi: 10.1371/journal.ppat.1012382 (PMC11265716; doi:10.1371/journal.ppat.1012382)
Supplement: S8 Table — EC50 values represent the weighted mean ± standard deviation of three biological replicates with each biological replicate comprised of two technical replicates. (DOCX) [file ppat.1012382.s008.docx]

| **Cell line** | **EC_50_ values, µM** |
| --- | --- |
| WT | 0.03 ± 0.005 (lower curve) |
|  | 5 ± 0.5 (upper curve) |
| P450R1 DKO | 7 ± 0.7 |
| CYP51 DKO | 5 ± 0.6 |
