# Supplementary figures and images for: Sterol 14-alpha demethylase (CYP51) activity in Leishmania donovani is likely dependent upon cytochrome P450 reductase 1
Source: PLoS Pathog. 2024 Jul 11;20(7):e1012382. doi: 10.1371/journal.ppat.1012382 (PMC11265716; doi:10.1371/journal.ppat.1012382)

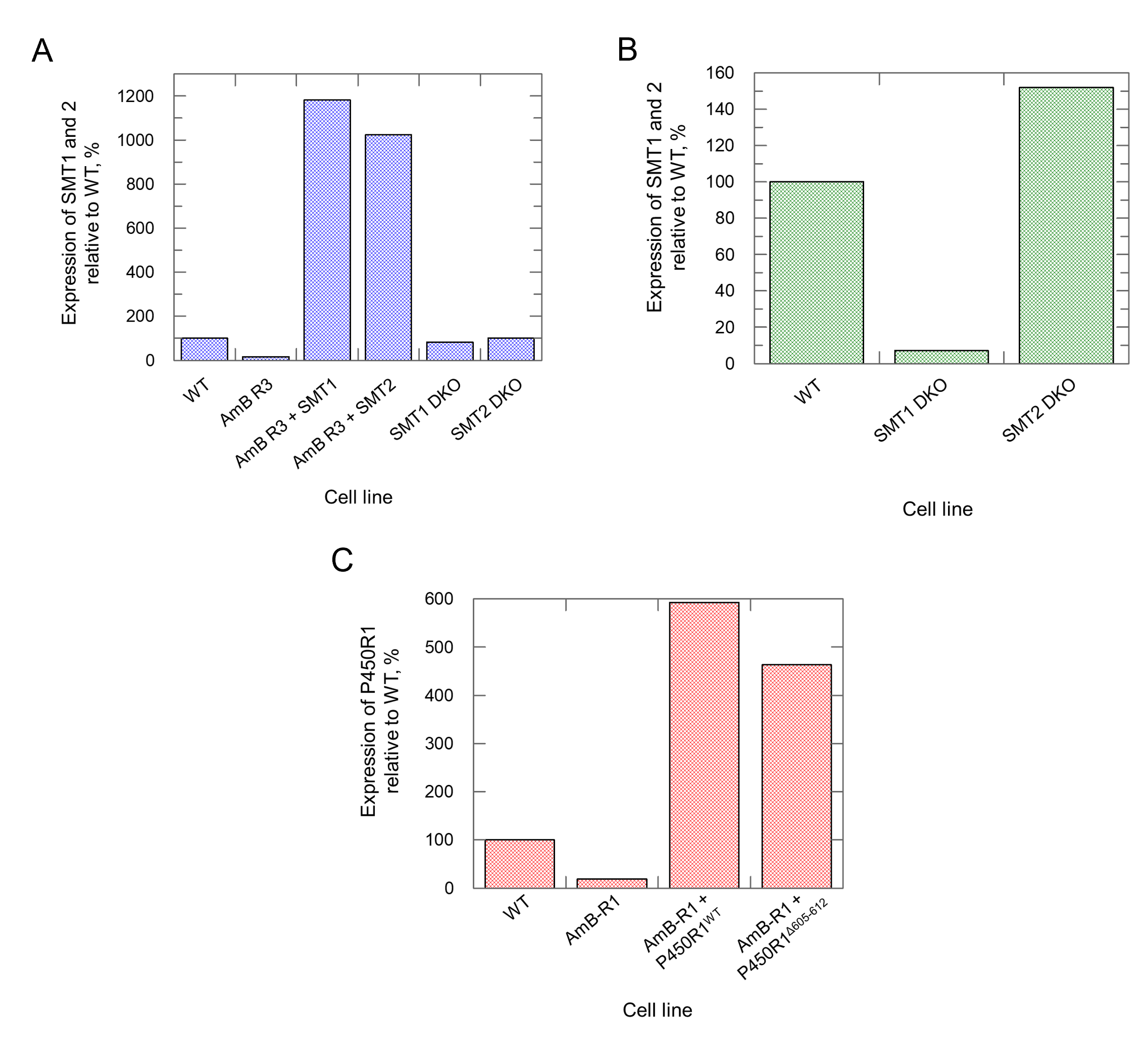

Supplement: S3 Fig — Protein levels (relative to WT) were determined by label free quantitation. SMT1/2 relative expression in promastigotes (A) and axenic amastigotes (B). (C) P450R1 protein levels (relative to WT) in promastigotes. Details of these analyses can be found in the Materials and Methods. (TIF) [file ppat.1012382.s011.tif]
